# Supplementary material for: High-dimensional topographic organization of visual features in the primate temporal lobe
Source: Nat Commun. 2023 Sep 22;14:5931. doi: 10.1038/s41467-023-41584-0 (PMC10517140; doi:10.1038/s41467-023-41584-0)
Supplement: Supplementary file 1 — Supplementary information [file 41467_2023_41584_MOESM1_ESM.pdf]

Supplementary Figures:

Figures S1-S11.

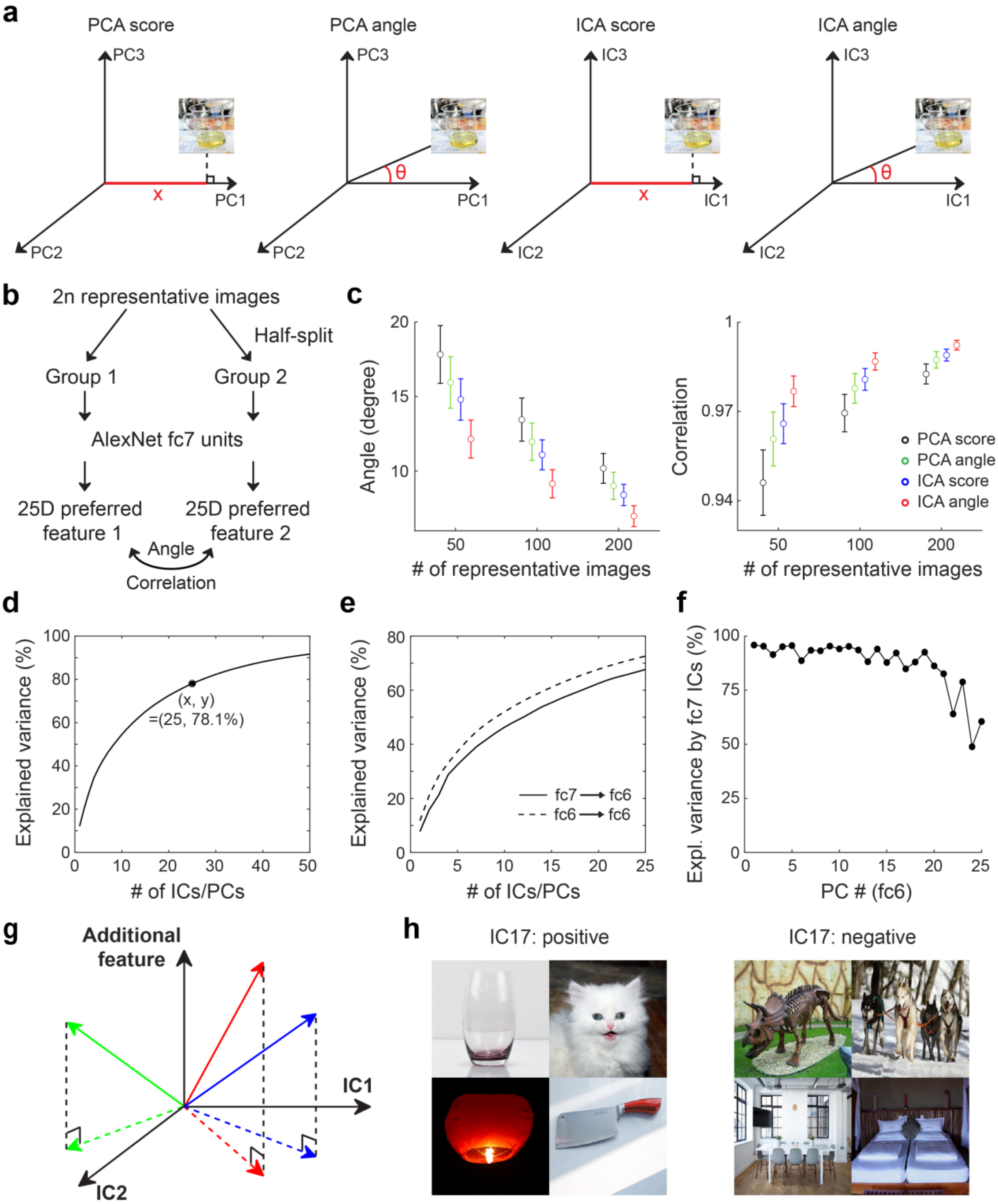

Figure S1. Comparing the reliability of AlexNet units' preferred features computed using different image selection procedures.

a) Two dimensionality reduction techniques, PCA and ICA, and two image selection criteria, one based on score and the other based on angle, were tested. PCA or ICA was performed on responses of units in layer fc7 of AlexNet. The PCA/ICA score of each image indicates the projection of its AlexNet representation onto a specific PC/IC axis, while the PCA/ICA angle indicates the angle between each image's AlexNet representation and a PC/IC axis. In the former case, images with the largest and smallest scores were selected as "positive" and "negative" representatives, while in the

latter case, images with the smallest and largest angles were selected (0°≤angle between vectors≤180°). b) After the representative images were selected using four procedures in (a), they were randomly split into two groups. All images were passed through AlexNet, and the responses of fc7 units were extracted. The 25D preferred feature of each unit was then computed by subtracting the mean response of "negative" representative images from that of "positive" representatives within each group. Preferred features of the two groups were compared using either the angle or the correlation between two vectors. c) The angle and the correlation between the two preferred features derived from half-splitting, computed using four image selection procedures and three numbers of selected images. Half-splitting was performed 1000 times, and the results were averaged for each unit. Error bar represents standard deviation of 4096 units in fc7. ICA angle was significantly more reliable than three other procedures in all cases ($p<0.001$, signed rank test, two-sided). d) The percentage of total variance in fc7 unit activations explained by different numbers of ICs or PCs extracted from fc7 units. e) The percentage of total variance in fc6 unit activations explained by different numbers of ICs or PCs extracted from units within the same layer (dashed line) or layer fc7 (solid line). f) The percentage of variance in fc6 PCs explained by the 25 fc7 ICs. Unit activations of layer fc6 and layer fc7 were used to explain the neural data in Bao et al. [1] and Cadieu et al. [2], respectively. g) In this diagram, we assume that the selectivity of each neuron can be represented by an axis in the space spanned by multiple features (the "axis" coding scheme [1,3]), which is indicated by a solid colored arrow. Dashed arrows indicate the projections of these axes (solid arrows) into the subspace spanned by the IC dimensions. An additional feature orthogonal to the ICs is indicated by the z-axis. h) Representative images for IC17. Same convention as Figure 1c. Note that due to copyright restrictions, the original ImageNet images in (a) and (h) are replaced with natural images from Pixabay (https://pixabay.com/). Source data are provided as a Source Data file.

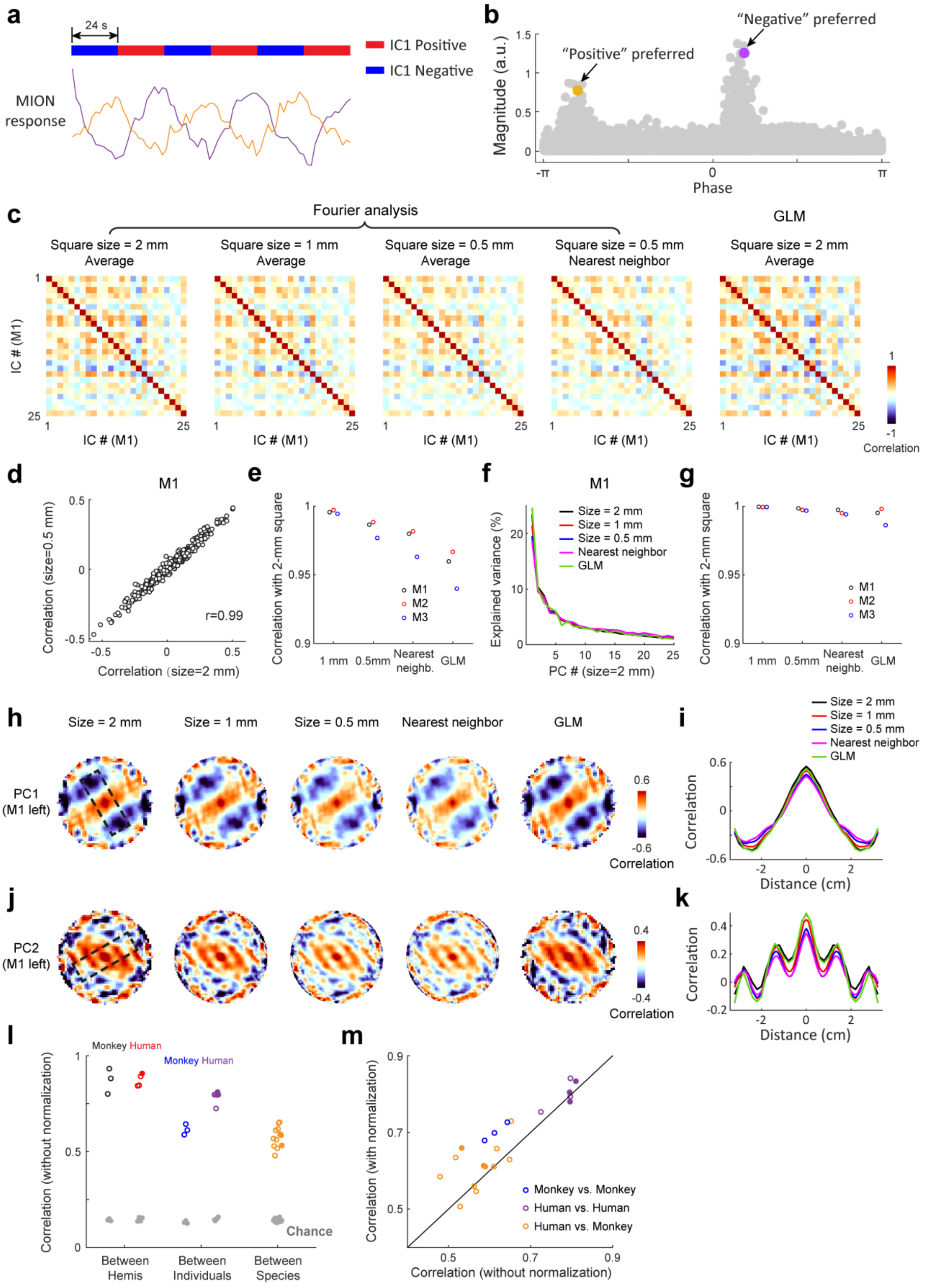

Figure S2. Comparing different ways of analyzing the feature maps.

a-b) Illustration of the Fourier analysis procedure. a) Average fMRI responses of two voxels in monkey 1 to six alternating blocks of positive and negative images in the first half of IC1 scans. Note that the response polarities are opposite to that of the conventional BOLD response due to the MION contrast agent intravenously injected. b) The magnitude and phase of each voxel in response to the alternation of positive and negative blocks, extracted using Fourier analysis, are plotted

against each other. The orange and purple dots, corresponding to the two voxels in (a), belong to two separate peaks of voxels that prefer “positive” and “negative” images, respectively. c) Population similarity matrices computed using three square sizes, two sampling approaches (average and nearest neighbor) and two analysis procedures (Fourier analysis and GLM). In the nearest neighbor approach, the feature of the vertex closest to the center of a square was assigned to that square. d) Correlation coefficients between ICs for two square sizes are plotted against each other. e) Consistency of similarity matrices, measured by Pearson correlation, between 2-mm square size and four other conditions. f) PCA was carried out on the feature maps computed using the 2-mm square size. Feature maps computed using all five different methods were projected onto the resulting PCs, and the percentages of variance explained by these PCs are plotted. g) Correlations between explained variances of 2-mm condition and those of other conditions. h) Spatial autocorrelation of monkey 1’s PC1 for the left hemisphere, computed using the five different approaches in (c). i) Autocorrelations within the rectangular box in (h) were projected onto its long edge. j-k) Same as (h)-(i), but for PC2. l) Same as Figure 2f, but without the normalization by the control block. For interhemispheric consistency, open and solid dots indicate male and female subjects, respectively. For intersubject consistency, open and solid dots indicate within-gender and between-gender comparisons, respectively. m) Consistency between subjects computed using feature maps before and after normalization are plotted against each other. The identity line is shown as a reference. Different colors indicate different combinations of species. For example, blue dots represent the intersubject consistency between three monkeys. Open and solid dots indicate within-gender and between-gender comparisons, respectively. Source data are provided as a Source Data file.

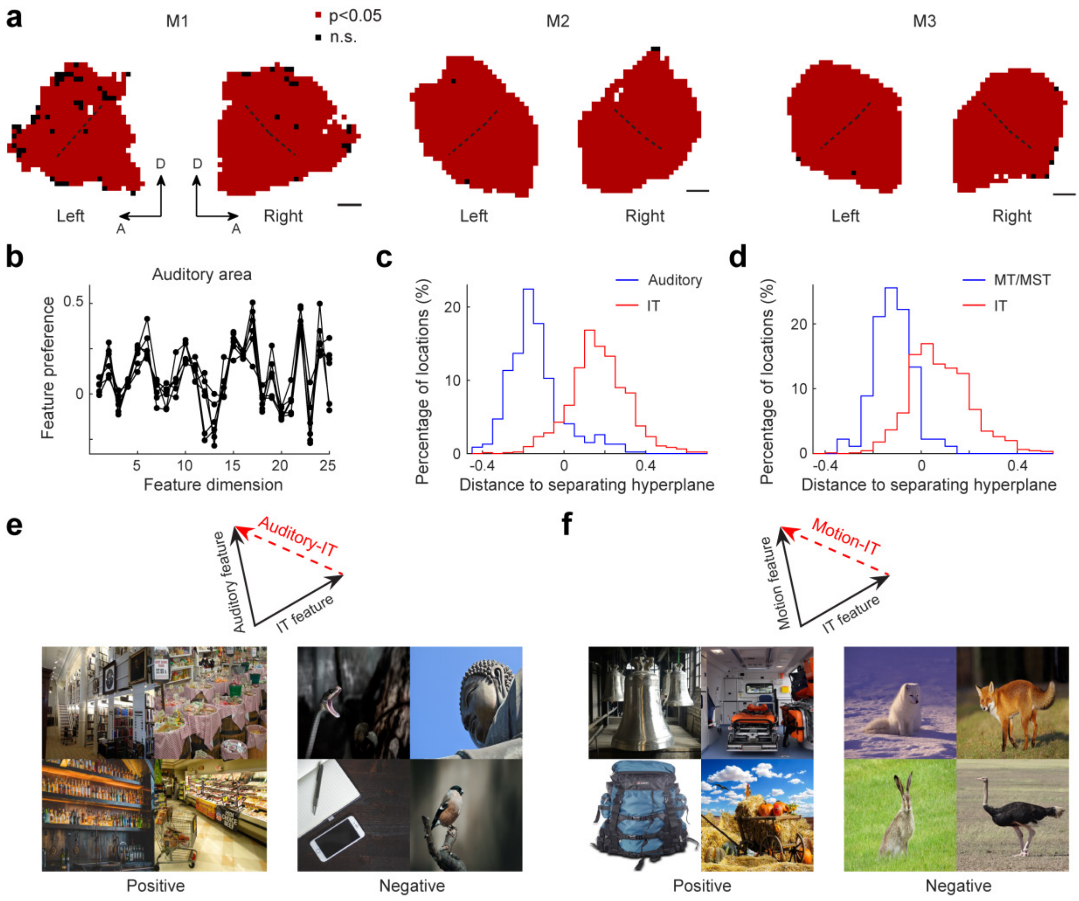

Figure S3. Neural tuning in the dorsal areas of the temporal lobe.

a) Reliability of neural tuning across the temporal lobe. All scans were randomly split into two halves and the similarity at each location between the 25-D preferred features of two halves was quantified using their cosine angle. We considered the tuning of a location to be significant if 95% of 500 iterations of random half-splitting resulted in a positive cosine angle. Dashed lines indicate the fundus of STS. b) Based on the CHARM atlas [4], preferred features of all squares within the auditory area of each hemisphere were averaged, resulting in six 25D preferred features, which are shown here. The average similarity between the average preferred features of all six hemispheres was 0.82 for auditory areas and 0.72 for motion areas ($p<0.01$ in both cases, tested by randomly shuffling the 25 IC dimensions for 2000 times). c) SVM models were trained to discriminate the preferred features of the auditory areas and IT (TE+TEO) of all three monkeys. Since there were fewer auditory features than IT features, the same number of features as the auditory features were randomly sampled from IT to train the classifier. The distribution of the distance to the optimal hyperplane for one iteration of random sampling is plotted. The average accuracy for 1000 iterations of random sampling is 87%. d) Same as (c), but between the motion areas (MT+MST) and IT. The average classification accuracy is 80%. e) The difference between the average preferred feature of the auditory areas and that of IT was computed. The positive and negative representative Images of the differential feature are shown. f) Same as (e), but between motion areas and IT. While the negative end contains mostly of isolated objects (IT), the images of the positive end are more crowded (dorsal areas). Note that due to copyright restrictions, the original ImageNet images in (e) and (f) are replaced with natural images from Pixabay (https://pixabay.com/). Source data are provided as a Source Data file.

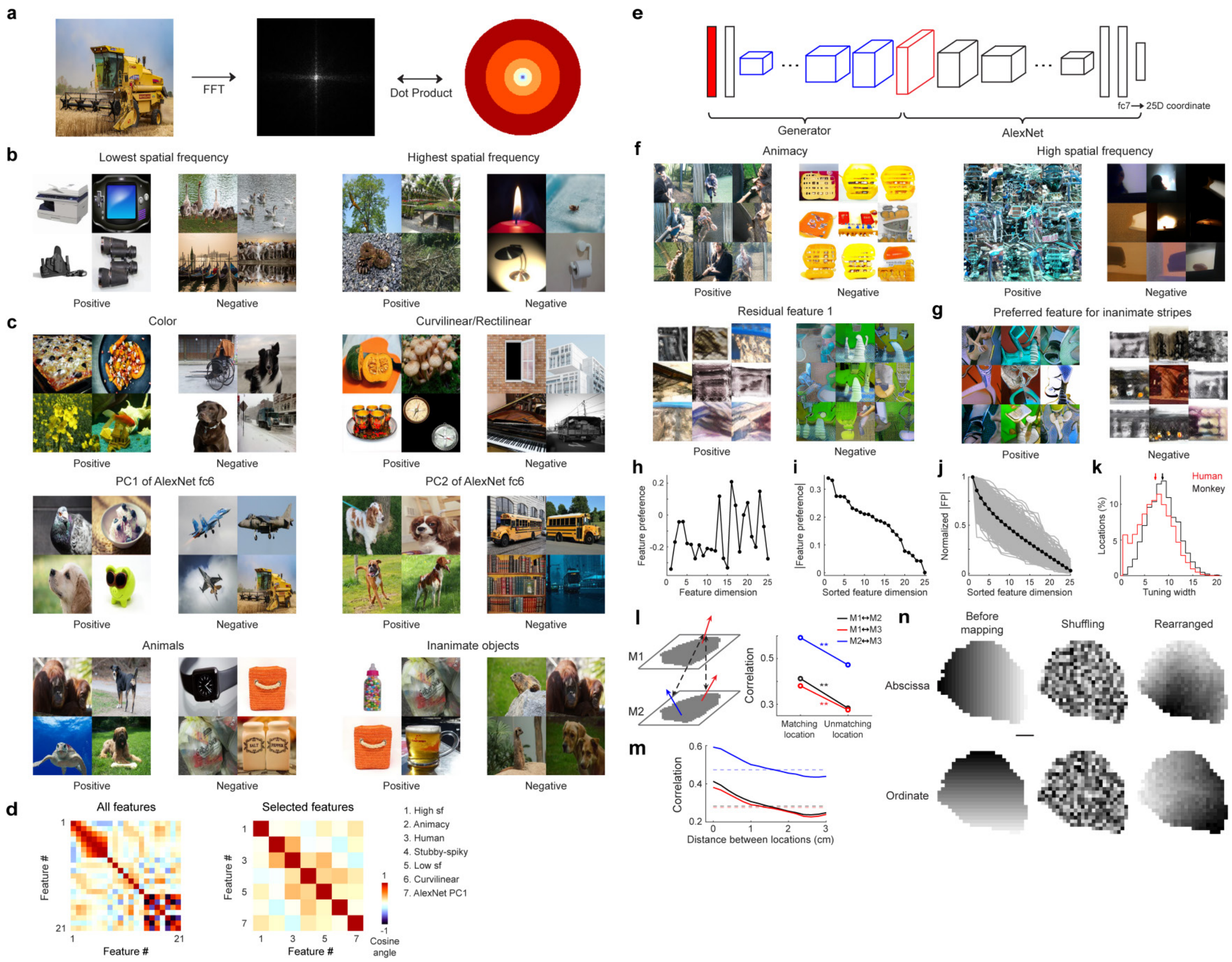

Figure S4. Representative images of interpretable features and preferred images synthesized using a generative model.

a) Using Matlab's fft2 function, two-dimensional Fourier transform of each image used in the fMRI experiment was obtained. Magnitudes within seven annuli of the power spectrum, ranging from low to high spatial frequencies, were averaged. These seven frequency bands

correspond to seven “spatial frequency” features. b) Representative images of the lowest and highest spatial frequency bands. c) Representative images of other features. d) Left: cosine angles between all 21 features in the 25D feature space. Right: cosine angles between seven selected features (red arrows in Figure 3c). e) Schematic for the GAN-based image synthesis procedure. We coupled a Generative Adversarial Network (BigGAN) as the prior along with AlexNet to synthesize new stimuli whose 25D coordinates matched that of a target feature. For details, see methods: Image synthesis using a generative model. f) Synthesized images for positive and negative directions of the three features in Figures 4b-e. g) Synthesized images for the preferred feature of inanimate stripes (the same feature as in Figure 6b). h) 25D preferred feature of one example location in M1’s temporal lobe. i) 25 preferences in (h) were sorted according to their absolute values. j) For each location, the absolute values of feature preferences were normalized so that the maximum was 1. Black line indicates the average of all locations in M1’s temporal lobe, and gray lines indicate individual locations. k) The number of dimensions with absolute values greater than half the maximum was used to define the feature tuning half-width for each location. The distributions for monkey and human data are plotted as histograms, with arrows indicating the averages (8.7 for monkey and 7.2 for human). l) After projecting feature maps of different monkeys onto a monkey template, we computed the correlation between 25D features of different monkeys at corresponding locations, and consistently found higher correlations between matching locations than between unmatching locations (right, ** indicates $p<0.01$, tested by randomly shuffling the location of squares while preserving the spatial proximity of nearby squares, see panel n for details of the shuffling procedure). m) Spatial cross-correlation between pairs of monkeys decreased monotonously as a function of distance between locations. Dotted lines indicate chance levels (same as unmatching locations in panel l). n) Illustration of the procedure used to create a random shuffling of squares on the flat map with the spatial proximity of nearby squares being preserved. We aimed to learn a mapping/transformation of the flap map onto itself, illustrated by plotting the abscissas and ordinates of the square locations before (left) and after the mapping (middle and right). We first shuffled all the squares randomly (middle), and then increased the continuity of the mapping step by step (right). For each step, we randomly selected a pair of squares and would swap their mapping if the continuity of the entire mapping increased after the swap—continuity was defined as the reciprocal of the total distance between the transformed locations of all pairs of originally neighboring squares (distance≤6 mm before the mapping). This procedure was repeated 8×10^4 times for the total distance to converge. Scale bar: 1 cm. Note that due to copyright restrictions, the original ImageNet images in (a)-(c) are replaced with natural images from Pixabay (https://pixabay.com/). Source data are provided as a Source Data file.

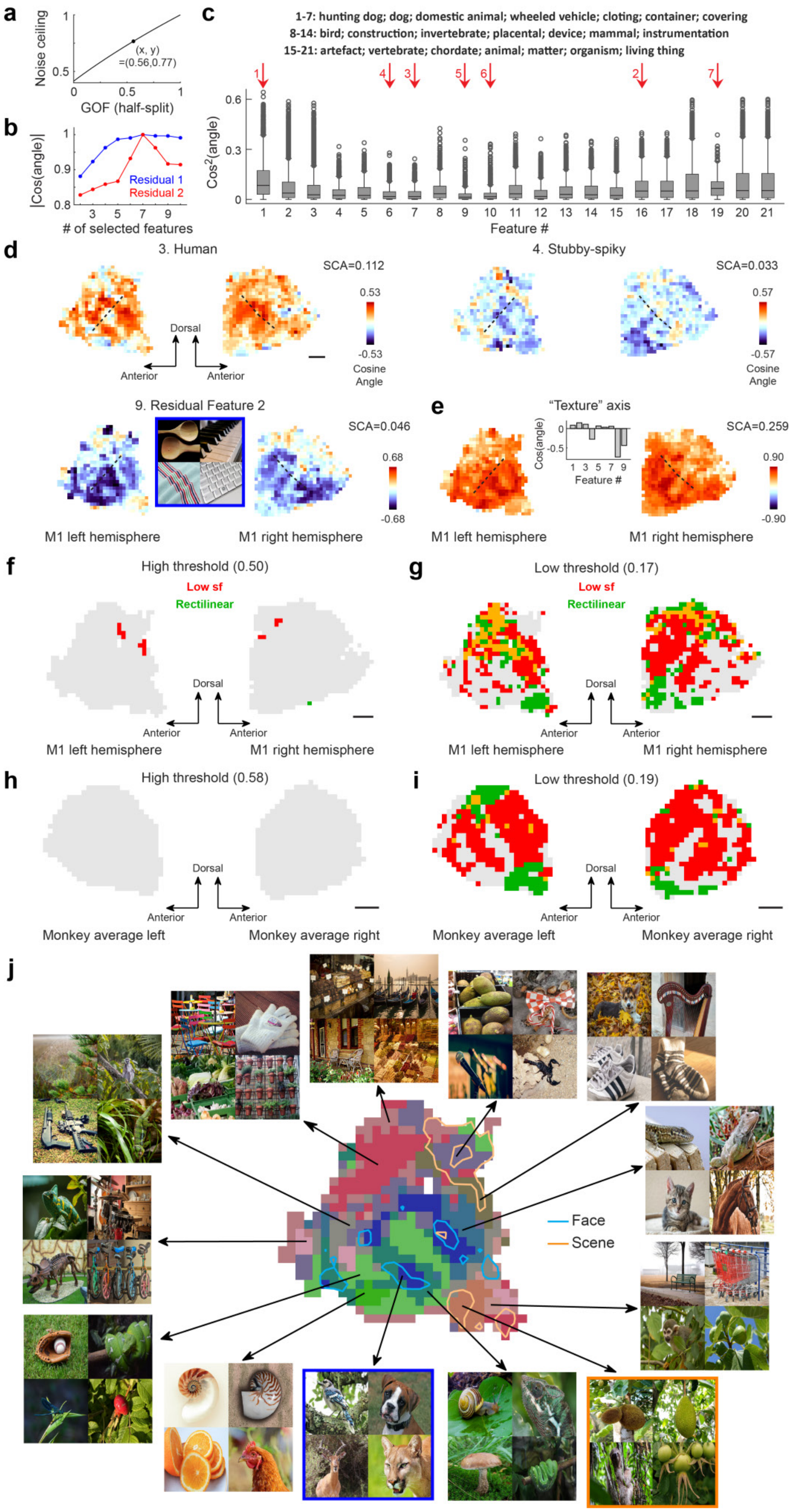

Figure S5. Additional analyses of the interpretable visual features.

a) Quantification of the noise ceiling. Different levels of random Gaussian noises were added to the 25D neural features of all three monkeys. To mimic the half-split analysis, two random simulations at the same noise level were generated, and the goodness-of-fit (GOF) was computed using $1 - ||f_1 - f_2||^2$, where $f_1$and f_2 were the 25D preferred features of two simulations at the same location. The noise ceiling was estimated using the same equation, but replacing f_1 or f_2 with the true noise-free 25D feature. The two indices were then averaged across locations and 100 repetitions of random simulations at each noise level and plotted against each other (black line). The half-split GOF for the neural data was estimated by 500 iterations of half-splitting (black dot). b) The residual features in Figures 3-4 were extracted from the neural features orthogonalized by the top seven selected features. To test the reliability of residual features, the same procedure was repeated, but with different numbers of selected features. The absolute cosine angles between the new residual features and the original ones based on seven selected features are shown. c) Similarity between the 25D neural features of three monkeys and 21 WordNet labels. Same convention as Figure 3c. The same feature selection procedure as in Figure 3 was followed. The seven most explanatory labels are (arrows): hunting dog, vertebrate, covering, container, construction, invertebrate, and matter. d) The 25D feature map of monkey 1 was projected onto three of the selected features. Same convention as Figure 4a. e) Same as (d), but for the "texture" axis in Figure 6b. The cosine angles between this axis and the 9 selected features in Figures 3-4 are shown in the inset. f and h) Same as Figures 4b and d, but for two different features. The thresholds for cosine angles were set at the same levels as in Figure 4 and are indicated in the brackets. g and i) Same as (f) and (h), but with lower thresholds (=1/3 of the original ones). j) Preferred images of M1's 13 clusters (see Figure 5). For each cluster, images with the smallest angles between their 25D coordinates and the average preferred feature of that cluster were selected. Some clusters represented easily interpretable features. For example, the dark blue cluster in the middle, a cluster composed of multiple smaller subregions, preferred images with animal faces clearly visible; the brown cluster on the ventral side preferred images with natural scenes, usually containing green plants. Separate experiments suggest these two clusters largely overlap with face patches and scene areas identified using conventional localizers (blue outlines: face, $p<0.002$; brown outlines: scene, $p<10^{-4}$; Student's t-test, two-tailed, not corrected for multiple comparisons). Scale bars: 1 cm. Note that due to copyright restrictions, the original ImageNet images in (d) and (j) are replaced with natural images from Pixabay (https://pixabay.com/). Source data are provided as a Source Data file.

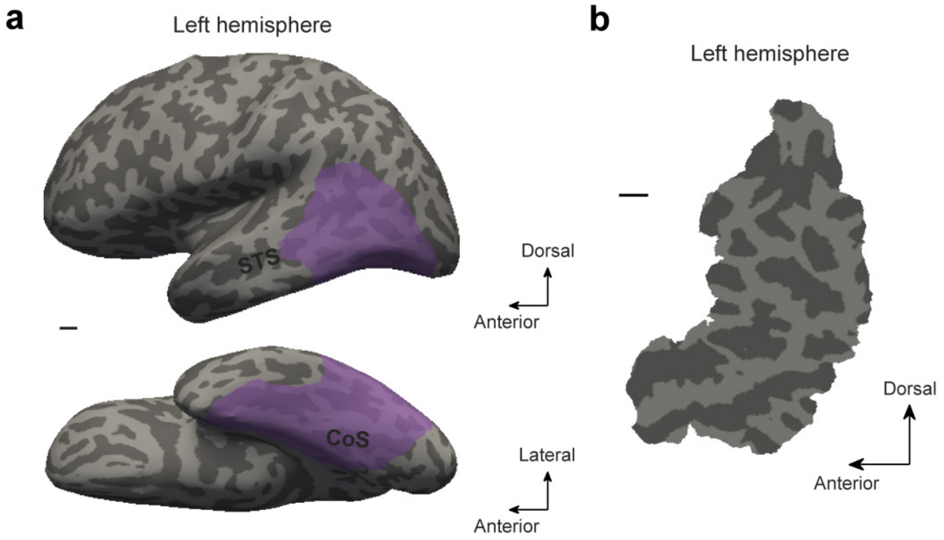

Figure S6. Inflated surface and flat map of a human subject.
a) The inflated surface of the left hemisphere of a human subject (H1), shown from two different perspectives. STS, superior temporal sulcus; CoS, collateral sulcus. b) The flat map of the left temporal lobe of the same subject (purple patches in panel a). Scale bars: 1cm.

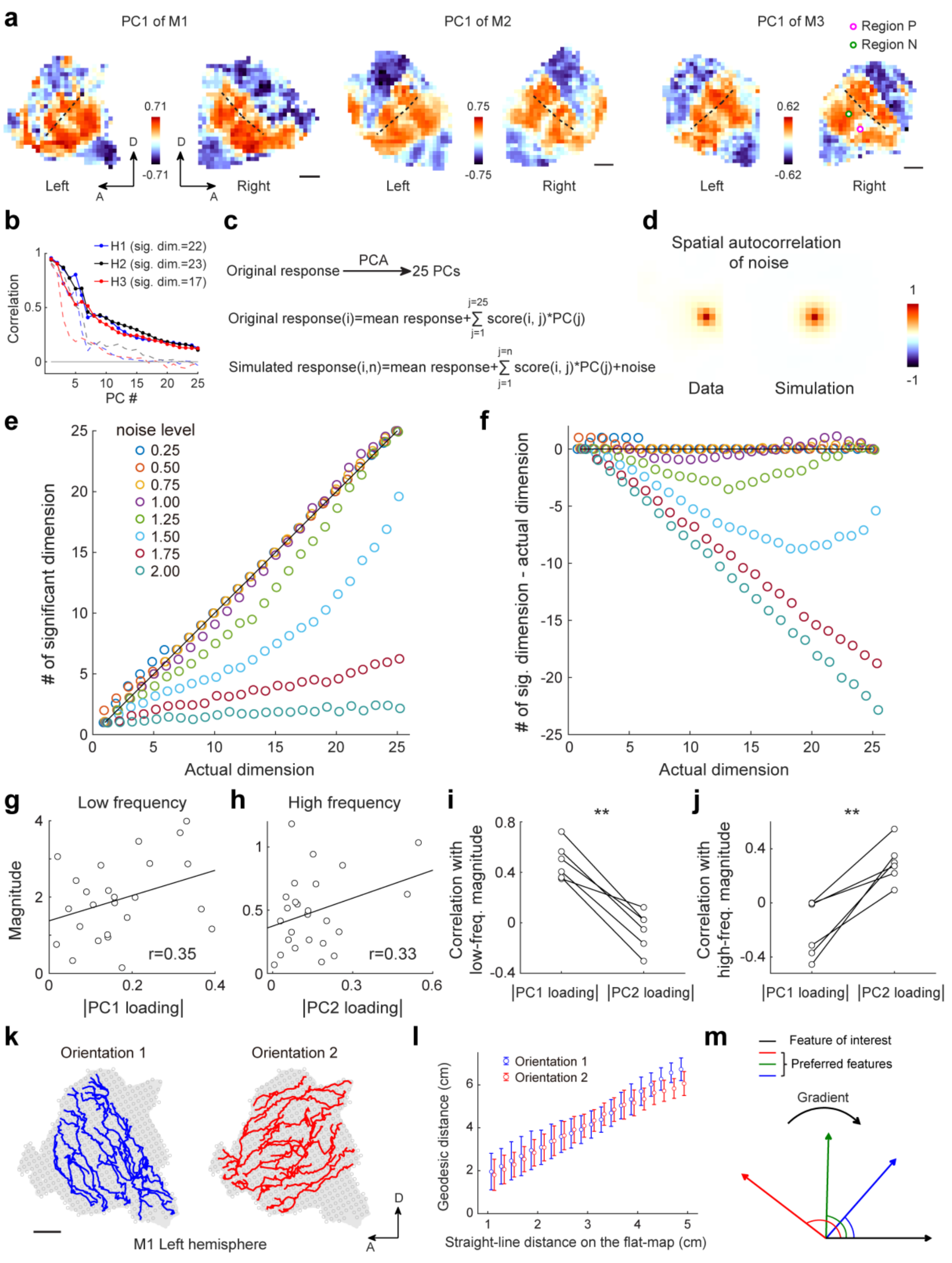

Figure S7. Additional analyses of the dimensionality and functional organization of feature maps.

a) Principal component analysis was separately carried out on the feature map of each monkey. Projections of the 25D feature map onto the 1st principal component are shown for three monkeys (dashed lines indicate the fundus of STS). Purple and green dots in the rightmost panel denote the two regions targeted for electrophysiological recordings (see Figures 6d and f). b) Results of half-split analyses for human subjects. Same convention as Figure 8b. For this analysis, we only included subjects who completed more than 8 scans per IC on average. H1 and H2 are male; H3 is female. c) The procedure for generating simulated feature maps. PCA was carried out

on the original feature map of M1. Top n PCs were used to reconstruct the responses, and a noise term was added, which was a standard Gaussian white noise spatially filtered to match the correlation structure of noise in real data. d) Spatial autocorrelation of noise for data and simulation. The full data set was randomly split into two halves, and the difference between the two resulting feature maps was used to compute the noise autocorrelation. 500 iterations of half-splitting were performed, and the resulting autocorrelations were averaged. e) The number of significant dimensions for the simulated data is plotted against actual dimensions, i.e., the number of PCs used for reconstruction, for multiple noise levels. At the noise level of 1, the average half-split correlation of the simulation roughly matched that of the data. f) The difference between the number of significant dimensions and the actual dimension of the simulation is plotted against the actual dimension. The difference hardly exceeds 1, suggesting the estimation is largely conservative. g-h) Autocorrelation analyses were carried out on individual IC dimensions for M1's left hemisphere. g) The low-frequency magnitude (see the left panel of Figure 8i) of each IC at the orientation indicated by the blue box in Figure 8e is plotted against its absolute loading for PC1. h) The high-frequency magnitude of each IC at the orientation indicated by the red box in Figure 8e is plotted against its absolute loading for PC2. The black lines indicate linear fits of the data. i) Correlations between low-frequency magnitudes of 25 IC dimensions and their absolute loadings for M1's PC1 and PC2 were computed and compared (**: $p<0.01$, paired t-test, two-tailed, n=6 hemispheres). j) Same as (i), but for high-frequency magnitudes. k) Example geodesic lines, roughly along two orthogonal orientations as in Figure 8e, were projected onto the flat map of M1's left hemisphere. Gray circles indicate the centers of 2 mm×2 mm squares aligned along the two orthogonal orientations (see Methods: Computing geodesic lines on the cortical surface). l) The relationship between geodesic distances and straight-line distances on the flap map for the two orientations (bin width=2 mm). Error bars represent SD. m) The definition of feature gradient in this paper. Each colored arrow indicates the 25D preferred feature of a brain region. The "gradient" refers to the gradual change in the angle between the preferred features and a given dimension (black arrow) across the cortical surface. Scale bars: 1 cm. Source data are provided as a Source Data file.

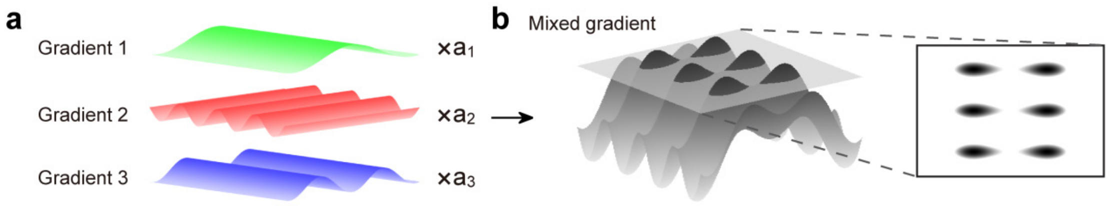

Figure S8. Feature gradients and discrete subregions.

a) The feature map can be viewed as a superposition of multiple feature gradients, each of which corresponds to a single "primary" feature and has a distinct orientation and spatial scale. b) The preference for a mix of the "primary" features would be distributed along a mixed gradient (left), and a threshold for brain regions with the strongest selectivity for that feature (the horizontal plane) might result in multiple separate subregions (right), just like the face patches identified in a conventional localizer experiment.

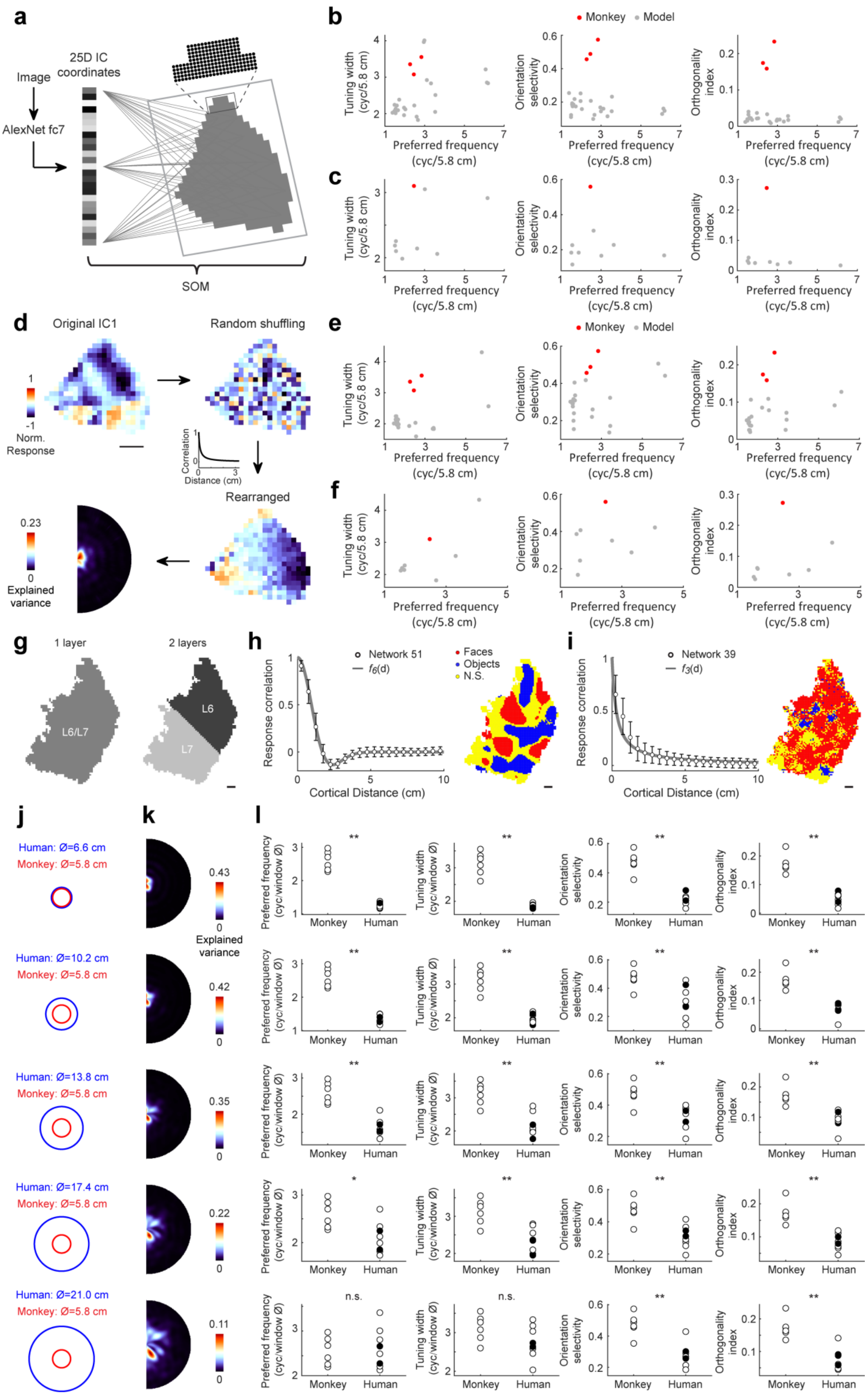

Figure S9. Additional analyses on neural network modeling.

a-c: Comparing self-organizing maps with the monkey feature map.

a) Model architecture. 25D IC coordinates extracted from AlexNet fc7 units were fed into a self-organizing map (SOM), which contained 6400 units arranged according to the shape of the monkey feature map. The topographic organization of units in SOM was constrained by a function of distance between units that controlled how the weight matrix between two layers was updated (see Methods: self-organizing map). We used either one of the seven functions shown in Figure 9g or a Gaussian function from a prior study [5] as the constraining function. Three random initializations were repeated for each function, yielding a total of 8×3=24 maps. The output feature map was then analyzed using the same procedures as in Figure 9. b-c) Frequency tuning width, orientation selectivity, and the orthogonality index are plotted against the preferred frequency for monkeys and SOMs. Same convention as Figures 9p-q. While the frequency tuning widths of the SOMs utilizing Gaussian constraining functions were comparable to those of the monkeys, the other two indices were considerably lower for all the SOMs.

d-f: Control models constructed by shuffling the monkey feature map to match spatial constraining functions.

d) Starting from the monkey feature map, we first shuffled all the squares randomly, and then increased the consistency between correlation structure of the shuffled map and a spatial constraining function step by step. For each step, we randomly selected a pair of squares and would swap their corresponding features if the correlation between the 25D features of a pair of squares became better predicted by one of the seven constraining functions in Figure 9g after the swap, quantified by the same spatial loss as the neural networks. The output feature map was then analyzed using the same procedures as in Figure 9. For each spatial constraining function, the procedure was repeated three times, resulting in a total of 7×3=21 maps. Scale bar: 1 cm. e-f) Frequency tuning width, orientation selectivity, and the orthogonality index are plotted against the preferred frequency for monkeys and control models. Same convention as Figures 9p-q.

g-i: Example results for topographic networks constrained by the shape of the human feature map.

g) The shape of the human feature map that was used to constrain the spatial arrangement of network units. Either one single layer (Left, L6 or L7) or two layers (Right, L6 and L7) were engaged in the feature map. See also Figure 9f. h) Same as Figures 9h and i, but for an example topographic network constrained by the shape of the human feature map. i) Same as (h), but for a different network. Scale bars: 1 cm.

j-l: In Figure 9s, the functional organization of human and monkey brains were compared using four different indices. One critical step in this comparison was to fit spatial autocorrelations within a certain circular window with sinusoidal functions. The diameter of the window differed between human and monkey brains due to overall brain sizes, but the exact ratio between the sizes of the two windows was hard to determine. Therefore, we varied the diameter of the window for human brains while fixing that for monkey brains to test the generality of the results in Figure 9s.

j) Blue and red circles illustrate the relative size of circular windows for analyzing human and monkey data, respectively. k) Same as Figure 9e, but for human data analyzed with the window sizes in (j). l) Same as Figure 9s, but for human data analyzed with the window sizes in (j). In almost all cases, monkey indices were significantly higher than human indices for frequency tuning width, orientation selectivity, and orthogonality index (*=p<0.05, **=p<0.01, Student's t-test, two-tailed). Open and solid dots indicate male and female subjects, respectively. Panels

showing significant differences between the two species remain significant when only males are considered. The results shown in Figures 9s are the same as the 4th row of (l) (Ø=17.4 cm for human data). This diameter was used in Figure 9 because the difference between the polar maps of human and monkey brains, i.e., the maps shown in (k) and Figure 9e, had the minimum vector length with this window size.

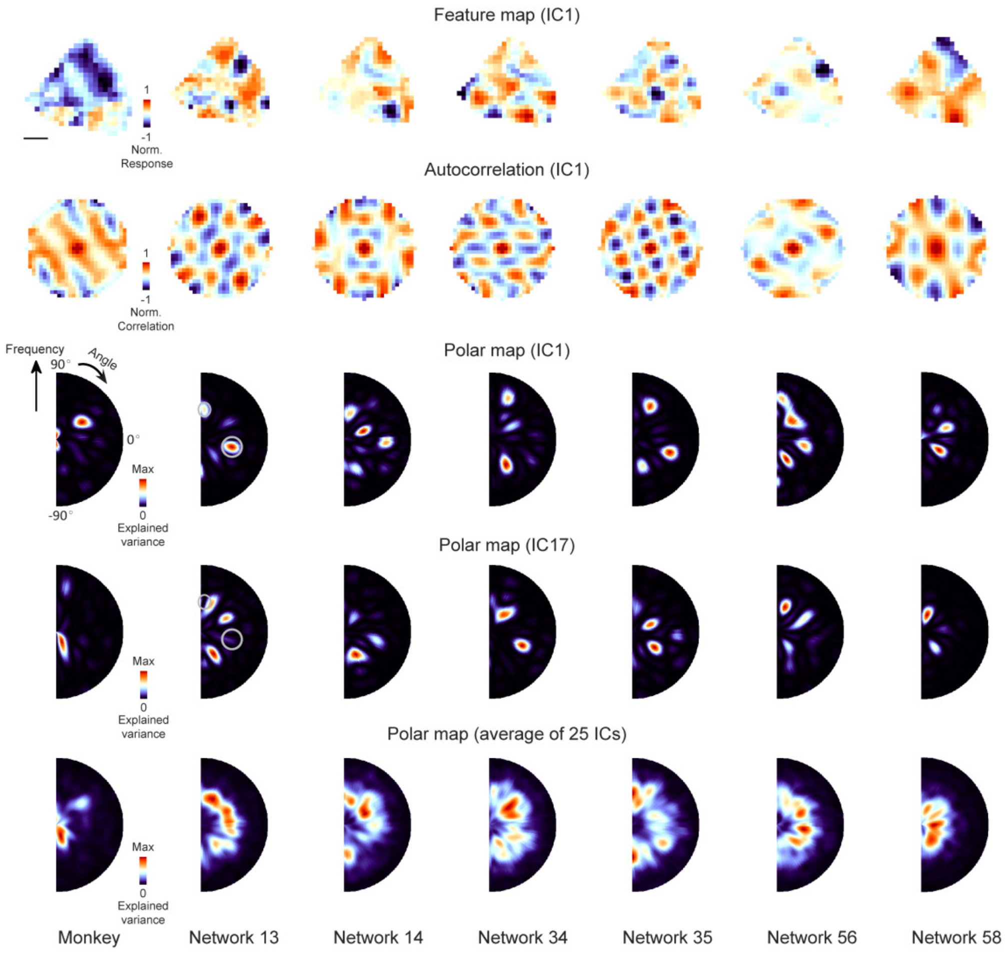

Figure S10. Comparison of monkey and model feature maps across multiple analysis steps. Each column indicates either the monkey or one of the network models in Figures 9f-q. Each row indicates an analysis step, from the original feature map to the autocorrelation map to the polar map as in Figure 9d. Maps in the top two rows are normalized so that the maximum absolute value is 1. The polar maps for IC1 and IC17 are shown, together with the average maps of 25 ICs. The gray circles in the 2nd column, 3rd row, outline the two peaks of the polar map. The same circles do not match well with the peaks of the map below in the 4th row. Scale bar: 1 cm.

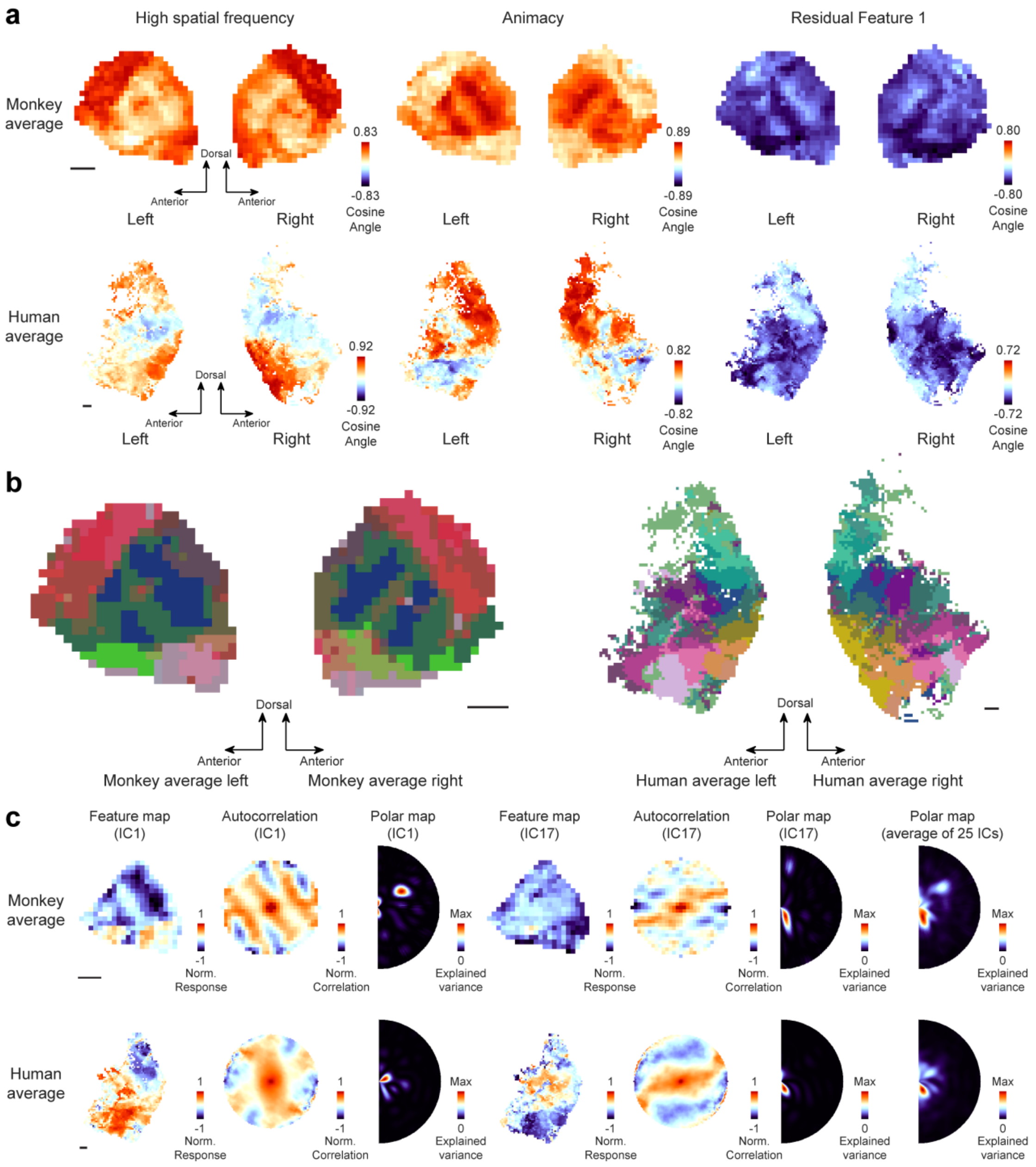

Figure S11. Additional comparisons of monkey and human feature maps.

a) The average feature maps of three monkeys (top) and four human subjects (bottom) were projected onto the "high sf" feature, the "animacy" feature, and the residual feature. The presence of negative projections onto the "animacy" feature for parts of the human map, but not the monkey map, is consistent with the stronger preference for inanimate objects in the human brain. b) Left: The clustering result for the average monkey feature map. Bayesian information criterion revealed the optimal number of clusters to be 15. Same convention as in Figure 5. Right: The clustering result for the average human feature map. The same number of clusters as in the monkey map was used for the human map. While blue and green stripes are visible in the monkey map, there is no such clear pattern in the human map. c) The original feature map, the autocorrelation map, and the polar map as in Figure 9d are shown for IC1 and IC17 of the monkey (top) and the human (bottom) feature maps. The average polar maps of all 25 ICs are also shown. Feature maps and autocorrelation maps are normalized so that the maximum

absolute value is 1. While the two ICs peak at different spatial frequencies in the monkey polar map, the two human maps peak at similar frequencies. Scale bars: 1 cm.

Supplementary References：

1 Bao, P., She, L., McGill, M. & Tsao, D. Y. A map of object space in primate inferotemporal cortex. *Nature* **583**, 103-108, doi:10.1038/s41586-020-2350-5 (2020).

2 Cadieu, C. F. *et al.* Deep neural networks rival the representation of primate IT cortex for core visual object recognition. *PLoS Comput Biol* **10**, e1003963, doi:10.1371/journal.pcbi.1003963 (2014).

3 Chang, L. & Tsao, D. Y. The Code for Facial Identity in the Primate Brain. *Cell* **169**, 1013-1028 e1014, doi:10.1016/j.cell.2017.05.011 (2017).

4 Jung, B. *et al.* A comprehensive macaque fMRI pipeline and hierarchical atlas. *Neuroimage* **235**, 117997, doi:10.1016/j.neuroimage.2021.117997 (2021).

5 Zhang, Y., Zhou, K., Bao, P. & Liu, J. Principles governing the topological organization of object selectivities in the ventral temporal cortex. *bioRxiv* **https://doi.org/10.1101/2021.09.15.460220** (2021).
