# Supplementary material for: High-dimensional topographic organization of visual features in the primate temporal lobe
Source: Nat Commun. 2023 Sep 22;14:5931. doi: 10.1038/s41467-023-41584-0 (PMC10517140; doi:10.1038/s41467-023-41584-0)
Supplement: Supplementary file 3 — Reporting Summary [file 41467_2023_41584_MOESM3_ESM.pdf]

nature portfolio

Corresponding author(s): Le Chang

Last updated by author(s): Aug 19, 2023

Reporting Summary

Nature Portfolio wishes to improve the reproducibility of the work that we publish. This form provides structure for consistency and transparency in reporting. For further information on Nature Portfolio policies, see our Editorial Policies and the Editorial Policy Checklist.

Statistics

For all statistical analyses, confirm that the following items are present in the figure legend, table legend, main text, or Methods section.

n/a	Confirmed	
☐	☒	The exact sample size (*n*) for each experimental group/condition, given as a discrete number and unit of measurement
☐	☒	A statement on whether measurements were taken from distinct samples or whether the same sample was measured repeatedly
☐	☒	The statistical test(s) used AND whether they are one- or two-sided<br>*Only common tests should be described solely by name; describe more complex techniques in the Methods section.*
☒	☐	A description of all covariates tested
☐	☒	A description of any assumptions or corrections, such as tests of normality and adjustment for multiple comparisons
☐	☒	A full description of the statistical parameters including central tendency (e.g. means) or other basic estimates (e.g. regression coefficient) AND variation (e.g. standard deviation) or associated estimates of uncertainty (e.g. confidence intervals)
☐	☒	For null hypothesis testing, the test statistic (e.g. *F*, *t*, *r*) with confidence intervals, effect sizes, degrees of freedom and *P* value noted<br>*Give P values as exact values whenever suitable.*
☒	☐	For Bayesian analysis, information on the choice of priors and Markov chain Monte Carlo settings
☒	☐	For hierarchical and complex designs, identification of the appropriate level for tests and full reporting of outcomes
☐	☒	Estimates of effect sizes (e.g. Cohen's *d*, Pearson's *r*), indicating how they were calculated

Our web collection on statistics for biologists contains articles on many of the points above.

Software and code

Policy information about availability of computer code

Data collection	Functional imaging data were collected by the Siemens Trio system (Siemens, Germany). Electrophysiological data were collected by the AlphaOmega system (AlphaOmega, Israel).
Data analysis	Functional imaging data were processed with Freesurfer and FSL. Custom codes written in MATLAB R2018 and Pytorch (1.7.1) were used for analysis, and are deposited at Zenodo (doi:10.5281/zenodo.8053796).

For manuscripts utilizing custom algorithms or software that are central to the research but not yet described in published literature, software must be made available to editors and reviewers. We strongly encourage code deposition in a community repository (e.g. GitHub). See the Nature Portfolio guidelines for submitting code & software for further information.

Data

Policy information about availability of data

All manuscripts must include a data availability statement. This statement should provide the following information, where applicable:

- Accession codes, unique identifiers, or web links for publicly available datasets
- A description of any restrictions on data availability
- For clinical datasets or third party data, please ensure that the statement adheres to our policy

Natural images used in the main stimulus set are available in the ImageNet (https://image-net.org/download.php). Face images used in the four-object-type

stimulus are available in the FEI database (https://fei.edu.br/~cet/facedatabase.html). The raw data supporting the current study are available under restricted access because of the size of the data and the complexity of its structure; access can be obtained by contacting Le Chang (lechang@ion.ac.cn).

Human research participants

Policy information about studies involving human research participants and Sex and Gender in Research.

Reporting on sex and gender	Eighteen healthy human subjects participated in the psychophysical (sex: four males and five females, age: between 23 and 26 years) and fMRI experiments (sex: five males and four females, age: between 23 and 38 years). All subjects had given written consent to the procedure in accordance with institutional guidelines and the Declaration of Helsinki. Subjects were compensated for their participation in the experiment: 1 Chinese yuan/minute for the psychophysical experiment and 2 Chinese yuan/minute for the fMRI experiment.<br>Sex/gender was not considered in the study design and, as a result, the number of subjects is insufficient for sex/gender-related analyses.<br>Sex of participants was determined based on self-report.
Population characteristics	Age of the nine subjects participating in the psychophysical experiment: mean=24.3 years; SD=1.2 year; min=23 years; max=26 years<br>Age of the nine subjects participating in the fMRI experiment: mean=27.0 years; SD=4.8 years; min=23 years; max=38 years
Recruitment	All participants are students and staffs of the Chinese Academy of Sciences recruited online. As a result, our subjects have higher levels of education than the general population. It's unknown whether visual processing depends on the level of education, but this could be a topic for a future study.
Ethics oversight	All experimental protocols were approved by the Biomedical Research Ethics Committee of the Institute of Neuroscience, Chinese Academy of Sciences.

Note that full information on the approval of the study protocol must also be provided in the manuscript.

Field-specific reporting

Please select the one below that is the best fit for your research. If you are not sure, read the appropriate sections before making your selection.

☒ Life sciences ☐ Behavioural & social sciences ☐ Ecological, evolutionary & environmental sciences

For a reference copy of the document with all sections, see nature.com/documents/nr-reporting-summary-flat.pdf

Life sciences study design

All studies must disclose on these points even when the disclosure is negative.

Sample size	Sample sizes were chosen in a manner commensurate with similar previous studies. Previous reports of fMRI experiments on macaques have proven that a sample size of three is sufficient (e.g., see Liu et al., Facial expressions evoke differential neural coupling in macaques, 2017; Taubert et al., Parallel processing of facial expression and head orientation in the macaque brain, 2020).
Data exclusions	For fMRI experiments, only scans with a total fixation time greater than 80% of the whole scan were included for further analyses. For electrophysiology, we recorded single-unit data from every neuron encountered. Only well-isolated units were considered for further analysis; otherwise, every neuron was included for analysis.
Replication	Results were replicated across individual subjects for each fMRI experiment. For reliability across subjects, see Figure 2f.<br>Our experiment contained 25 stimulus conditions (25 ICs), and we obtained ~13 independent scans per condition for each subject. Reliability across scans was confirmed using a half-split approach (see Figure 8b and Figure S3a).
Randomization	The stimuli were shown in a random order.
Blinding	The investigators were not blinded. This study is essentially a within-subjects design, in which responses to two groups of images (positive and negative representative images) are compared for each stimulus condition. The presentation of the images is controlled by the computer and cannot be interfered with by the investigators.

Reporting for specific materials, systems and methods

We require information from authors about some types of materials, experimental systems and methods used in many studies. Here, indicate whether each material, system or method listed is relevant to your study. If you are not sure if a list item applies to your research, read the appropriate section before selecting a response.

Materials & experimental systems

n/a	Involved in the study
☒	☐ Antibodies
☒	☐ Eukaryotic cell lines
☒	☐ Palaeontology and archaeology
☐	☒ Animals and other organisms
☒	☐ Clinical data
☒	☐ Dual use research of concern

Methods

n/a	Involved in the study
☒	☐ ChIP-seq
☒	☐ Flow cytometry
☐	☒ MRI-based neuroimaging

Animals and other research organisms

Policy information about studies involving animals; ARRIVE guidelines recommended for reporting animal research, and Sex and Gender in Research

Laboratory animals	Three adult rhesus macaques (Macaca mulatta, all males, 4-6 years old, weighing 5–8 kg) were used in this study.
Wild animals	The study did not involve wild animals.
Reporting on sex	All animal subjects are male.<br>Sex was not considered in the study design.
Field-collected samples	The study did not involve field-collected samples.
Ethics oversight	All experimental procedures were approved by the Biomedical Research Ethics Committee of the Institute of Neuroscience, Chinese Academy of Sciences, and were in accordance with the National Institutes of Health Guide for the Care and Use of Laboratory Animals.

Note that full information on the approval of the study protocol must also be provided in the manuscript.

Magnetic resonance imaging

Experimental design

Design type	Block design
Design specifications	During the fMRI experiment, stimuli were presented in 24 s blocks. Each scan contained 17 blocks and lasted 408 s.
Behavioral performance measures	The subject's eye position was monitored using an infrared eye tracking system (EyeLink 1000 Infrared Eye Tracker, SR Research, Mississauga, Ontario, Canada). For animal experiments, juice reward was delivered every 2–4 s if fixation was properly maintained (within a 2.5-degree-diameter circular window).

Acquisition

Imaging type(s)	Functional and anatomical imaging
Field strength	3 Tesla
Sequence & imaging parameters	Human study:<br>Whole-brain fMRI data were collected using a gradient-echo echo-planar imaging (EPI) sequence (TR = 2000 ms; TE = 30 ms; flip angle = 90°; slices = 50; matrix = 80 × 80; field of view = 240 mm × 240 mm; 3 mm × 3 mm in plane resolution; slice thickness = 3 mm; GRAPPA factor = 1). A pair of gradient echo images (echo time: 8 and 10.46 ms) with the same orientation and resolution as EPI images were acquired to generate a field map for distortion correction of EPI images. High-resolution T1-weighted anatomical images were acquired using a MPRAGE sequence (TR = 2300 ms; TE = 3 ms; inversion time = 1000 ms; flip angle = 9°; acquisition voxel size = 0.5 mm × 0.5 mm × 0.5 mm; 176 sagittal slices).<br><br>Animal study:<br>Whole-brain fMRI data were collected using a gradient-echo echo-planar imaging (EPI) sequence (TR = 2000 ms; TE = 24 ms; flip angle = 80°; slices = 28; matrix = 64 × 64; field of view = 96 mm × 96 mm; 1.5 mm × 1.5 mm in plane resolution; slice thickness = 2 mm; GRAPPA factor = 2). A pair of gradient echo images (echo time: 3.4 and 5.86 ms) with the same orientation and resolution as EPI images were acquired to generate a field map for distortion correction of EPI images. High-resolution T1-weighted anatomical images were acquired using a MPRAGE sequence (TR = 2300 ms; TE = 2.7 ms; inversion time = 1100 ms; flip angle = 9°; acquisition voxel size = 0.5 mm × 0.5 mm × 0.5 mm; 224 horizontal slices).
Area of acquisition	Whole brain

Diffusion MRI ☐ Used ☒ Not used

Preprocessing

Preprocessing software	Analysis of functional volumes was performed using the FreeSurfer Functional Analysis Stream. Volumes were corrected for motion and undistorted based on acquired field map. Surface reconstruction based on anatomical volumes was performed using FreeSurfer after skull stripping using FSL's Brain Extraction Tool (University of Oxford). After applying these tools, segmentation was further refined manually.
Normalization	To directly compare the functional organization of visual features between subjects, each subject's brain was registered to a common template of the species to which it belongs, using FreeSurfer's mri_cvs_register function.
Normalization template	For monkey and human templates, we used NMT v2 and CVS atlas, respectively.
Noise and artifact removal	We removed the linear or quadratic trends in the timeseries.
Volume censoring	Volume censoring was not applied.

Statistical modeling & inference

Model type and settings	We used only first-level analysis.
Effect(s) tested	We ran t-tests between different conditions within each single subject.

Specify type of analysis: ☒ Whole brain ☐ ROI-based ☐ Both

Statistic type for inference (See Eklund et al. 2016)	All the analyses were done using voxel-wise inference.
Correction	We did not apply any multiple-comparison correction in the fMRI imaging analysis.

Models & analysis

n/a	Involved in the study
☒	☐ Functional and/or effective connectivity
☒	☐ Graph analysis
☒	☐ Multivariate modeling or predictive analysis
